# Hydrogen Peroxide Diffusion and Optical Response in a Universal Composite Resin: A Spectrophotometric In Vitro Investigation with Clinical Implications

**DOI:** 10.4317/jced.63941

**Published:** 2026-04-25

**Authors:** Francesca Zotti, Enrique-Antonio Espinoza, Giorgia Lanzaretti, Marius Bud

**Affiliations:** 1 Department of Surgical Sciences, Pediatrics and Gynecology, University of Verona, P.le L.A.Scuro, 10, 37134 Verona, Italy; 2 Private practice, Trento, Italy; 3 Private practice, Vicenza, Italy; 4 “Iuliu Hațieganu” University of Medicine and Pharmacy, Motilor Str., 33, Cluj-Napoca, Romania

## Abstract

**Background:**

The aim of this in vitro study was to evaluate the extent to which a hydrogen peroxide-based whitening agent penetrates a universal composite resin and induces optical changes detectable by spectrophotometry.

**Material and Methods:**

60 rectangular-shaped composite specimens were fabricated and finished using a standardized protocol, then divided into two groups based on the polishing method used: 30 specimens were polished with a single-step polishing system (Enhance), and the remaining specimens were polished with a two-step polishing system(Astropol). Each group was further divided into two subgroups based on the ageing solution in which they were submerged after polishing: red wine (study groups) or physiological solution (control groups). Specimens then underwent ageing and were exposed to a 40% peroxide gel at predefined intervals. Color measurements (L*, a*, b*) were recorded before and after treatment and overall color difference (Eab*) was computed according to the CIE L*a*b* formula. Statistical analysis was performed using software and applying Wilcoxon's W test and Welch's t test.

**Results:**

In both study groups, a statistically significant difference in Eab* values was found between the measurement points after bleaching (p &lt; 0.05). Between-group comparisons showed statistically significant differences in lateral Eab* after bleaching (p &lt; 0.05). All measurement points achieved final Eab* values 3.3 following whitening treatment.

**Conclusions:**

Within the limitations of this study, hydrogen peroxide-based systems appear capable of diffusing into universal composite resins and altering their optical properties. Furthermore, the bleaching effect appears to be greater in specimens polished using a single-step method.

## Introduction

Aesthetic demands in contemporary dentistry have increased the popularity of vital tooth whitening procedures, both at home and in-office protocols (1). Whitening gels based on hydrogen peroxide (HP) or carbamide peroxide deliver reactive oxygen species capable of cleaving organic chromophores within dental tissues, thereby increasing lightness and reducing chromatic saturation (1 - 3). Although efficacy and safety have been widely documented in enamel and dentin, the interaction between whitening agents and existing composite restorations remains a matter of practical concern (4 - 6). Composite resins-consisting of a dimethacrylate matrix reinforced with inorganic fillers and a silane coupling system-exhibit overall color stability in routine clinical service; yet, microstructural aspects such as resin composition, filler load, degree of conversion, and surface finishing may modulate their susceptibility to oxidation and water sorption (7 - 10). Several reports have suggested that bleaching agents, depending on exposure time and formulation, may be able to improve optical parameters of composite restorations, or modify their surface characteristics (5 , 11 - 15). From a clinical standpoint, patients may express esthetic dissatisfaction due to shade mismatch of existing anterior composite restorations-which could have been exposed to chromogenic substances; clinicians could therefore evaluate the possibility of whitening treatment on discolored composite restorations (14 , 16 - 18). The present in vitro investigation was designed with these aims: 1. Evaluation, by means of spectrophotometric analysis, of penetration of a whitening agent into a block of universal composite resin previously immersed in red wine, and its ability to modify the material's color. 2. To evaluate, through spectrophotometry, whether a difference exists in the penetration of a whitening agent into a universal composite resin block that has been finished and polished using two different techniques.

## Material and Methods

**Figure 1:**
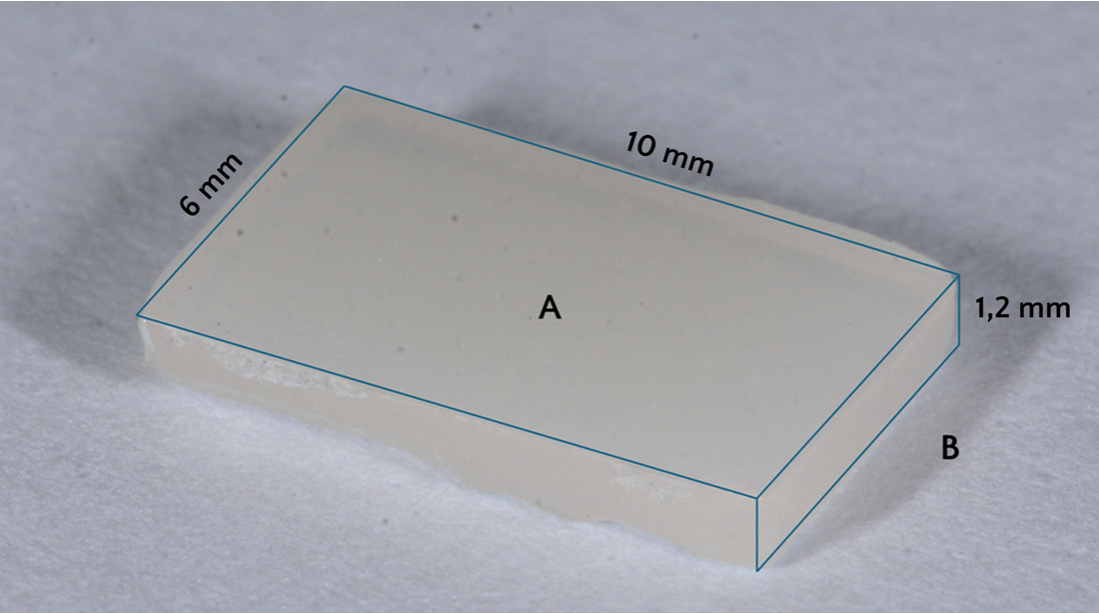
Naming of the sample surfaces, indicated in the figure as “A” and “B”.

The sample size calculation for this study, based on a review of the recent literature in the field (19), was performed using a dedicated software (G*Power, version 3.1.9.7 for Windows 11, Heinrich-Heine University, Düsseldorf, Germany). Parameters were set as follows: effect size = 1, = 0.05, and statistical power = 0.85. To detect a statistically significant difference among groups, the minimum required number of specimens was 19. To maintain adequate statistical power and to compensate for potential sample loss due to procedural errors, the sample size was increased by 20%, resulting in 23 specimens per study group. Additionally, two control groups consisting of seven specimens were included. In total, 60 specimens were prepared and divided into four groups as follows: Astropol Group : 30 specimens polished using a multi-step technique divided as follows: o 23 specimens exposed to red wine after polishing (Astropol study group - AG) o 7 specimens exposed to physiological solution after polishing (Astropol control Group - AGC) Enhance Group: 30 specimens polished using a single-step technique divided as follows: o 23 specimens exposed to red wine after polishing (Enhance study group - EG) o 7 specimens exposed to physiological solution after polishing (Enhance control group - EGC) Control groups were smaller in size, as they were intended to provide a baseline reference condition with expected minimal variability. Sample size calculation was therefore primarily based on experimental groups. - Specimen Fabrication Composite specimens were fabricated using 3D-printed ASA trays containing standardized rectangular molds (10 × 6 × 1.2 mm). These were filled with Omnichroma composite (Tokuyama Dental, Japan), covered between two transparent plates to obtain smooth parallel surfaces, and light-cured for 20 s per side (40 s total) with an LED curing unit (Woodpecker iLed MAX). After polymerization, the transparent plates were separated and the specimens were carefully removed to avoid fractures, ensuring uniform geometry and curing among samples. Specimens were included if they exhibited uniform, smooth surfaces and were free from internal defects (e.g. air bubbles) and external defects (e.g. fractures, chipped or incomplete margins, stains) that could affect color measurements. All specimens met the inclusion criteria for this study. A layer of transparent nail varnish (Lasting Color, PUPA, Milan, Italy) was applied to all specimen surfaces except two. The larger non-varnished surface was coded as A, and the smaller lateral one as B (Fig. 1).

1

These two uncoated areas, referred to as free surfaces, were the only parts of each specimen left exposed. The specimens were left in a well-ventilated environment for 12 hours to allow the varnish to harden, and excess material was carefully removed from the free surfaces using a curette. The specimens were manually shuffled by one operator and randomly assigned to four groups, and their free surfaces were polished using two different techniques (Table 1): AG and AGC groups: polished with the Astropol® system, first using the F tip and then the P tip, applying light constant pressure for 10 s per specimen (10).

Table 1

EG and EGC groups: polished with the Enhance® PoGo® system, using the PoGo® tip with progressively decreasing pressure for 10 s per specimen, following the manufacturer's instructions (10). After polishing, all specimens were stored in physiological solution at room temperature and low light for 24 hours to complete the polymerization process and promote resin hydration. - Color Measurement Protocol The color of the composite specimens was objectively measured using a SpectroShade Micro® spectrophotometer (MHT, 2006; MHT S.r.l., Oxnard, CA, USA) at five different time-points (Table 2).

Table 2

**Figure 2:**
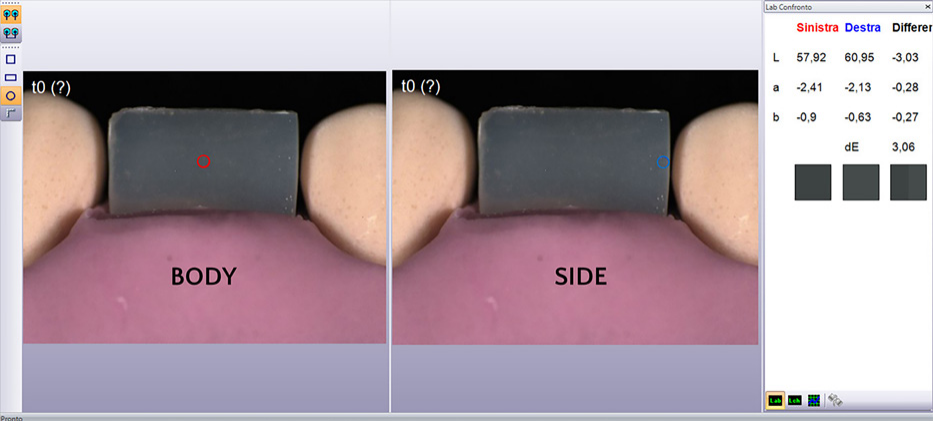
Figure exported from “MHT SpectroShade” color analysis software. The BODY point is marked with a red selector, and the SIDE point with a blue selector. The right-hand column displays the L*, a* and b* color coordinates for both points, as well as their resulting color difference.

The color assessment was performed once per specimen according to the following standardized parameters: The spectrophotometer sensor was positioned in a low-light environment created by a folded black cardboard enclosure, minimizing ambient light interference and providing a uniform black background to simulate intraoral conditions. A hard pink silicone support (Zetalabor Putty Hard 80 Shore A, Zhermack Dental, Germany) was used to simulate gingival tissues, featuring a central groove and two lateral grooves. Two resin teeth (shade A1, VITA scale) were placed within the lateral grooves of the silicone support to simulate adjacent dental elements. Each composite specimen, rinsed for 10 seconds and gently dried with absorbent paper, was positioned in the central groove of the support, with surface A oriented perpendicularly toward the spectrophotometer's sensor and surface B facing right. The spectrophotometer was calibrated prior to sample measurements and then recalibrated between the two main experimental groups; all measurements were performed by the same operator and no average of measurement was calculated. Color measurements were recorded at two specific points using the device software: the BODY point, located at the center of surface A, and the SIDE point, located at the center of the edge of surface A contiguous with surface B (Fig. 2).

2

The color coordinates (L*, a*, b*) obtained from the CIELAB color system were transcribed into a spreadsheet for data storage and subsequent statistical analysis, (Table 2). - Immersion Protocol and Color Measurements Over Time The initial color of each composite specimen was measured using a spectrophotometer after the hydration period (T0), as described before. Each specimen was then placed individually into small silicone containers (internal dimensions 10 × 10 × 10 mm), ensuring that the free surfaces remained unobstructed. The wells containing the AGC and EGC specimens were filled with physiological solution (NaCl 0.9%, Sodium Chloride, B. Braun, Melsungen, Germany), while those containing the AG and EG specimens were filled with red wine. Using a syringe with a needle to prevent air bubble formation, the liquids were slowly introduced into the wells until the specimens were fully submerged, guaranteeing uniform exposure of all free surfaces to the solution. The composite specimens were stored in their respective solutions, in darkness and at room temperature, for a total of 14 days. Every 24 hours, throughout the entire experimental period, the solutions were renewed, and each specimen was rinsed with water for 10 seconds. During the exposure period, intermediate color measurements were performed by spectrophotometry after 7 days (T1) and 14 days (T2), following the same procedure described previously. According to the literature, 24 hours of uninterrupted in-vitro exposure corresponds approximately to one month of intraoral exposure (19); therefore, the total exposure time in this in vitro study simulated approximately one year of clinical use. At the end of the 14-day exposure period, all specimens were immersed in physiological solution for an additional 24 hours to simulate a washout phase without chromogenic substances. After this interval, an additional color measurement (T3) was performed using the spectrophotometer. - Whitening Protocol The B surface of all specimens was subjected to a whitening procedure by applying a 1 mm-thick layer of 40% hydrogen peroxide gel (Opalescence Boost, Ultradent Products Inc., Milan, Italy) for 20 minutes, following the manufacturer's instructions. The bleaching gel was carefully applied to cover only surface B, ensuring that all other specimen surfaces remained unaffected by the whitening treatment. After the exposure period, each specimen was thoroughly rinsed with running water for 10 seconds, gently blotted dry with absorbent paper, and then underwent a final color measurement (T4) using the spectrophotometer. - Color-change Calculation The Eab* values between the different time-points (T0, T1, T2, T3, and T4), at the two measurement sites of each specimen (BODY and SIDE), were calculated using the following formula: Eab* = ((L*)2 + (a*)2 + (b*)2). Specifically, the following parameters were defined: Eab* B1: color difference at the BODY point between T0 and T1; Eab* B2: color difference at the BODY point between T0 and T2; Eab* B3: color difference at the BODY point between T0 and T3; Eab* B4: color difference at the BODY point between T0 and T4; Eab* B5: color difference at the BODY point between T3 and T4, indicating the bleaching effectiveness; Eab* S1: color difference at the SIDE point between T0 and T1; Eab* S2: color difference at the SIDE point between T0 and T2; Eab* S3: color difference at the SIDE point between T0 and T3; Eab* S4: color difference at the SIDE point between T0 and T4; Eab* S5: color difference at the SIDE point between T3 and T4, also indicating the bleaching effectiveness. - Statistical Analysis The data were analyzed using statistical software (Jamovi, version 2.6.26 for Windows, Sydney, Australia) to achieve the following objectives: To obtain descriptive statistics (mean and standard deviation) of the Eab* values at the different study times for all four groups; To assess whether there was a statistically significant difference in bleaching effectiveness (Eab*5) between the BODY and SIDE points, using the Wilcoxon W test; To assess whether there was a statistically significant difference in the ability to return to the original color (Eab*4) between the BODY and SIDE points, using the Wilcoxon W test; To evaluate whether there was a statistically significant difference in bleaching effectiveness at the SIDE point (Eab*S5) between the AG and EG groups, using the Welch's t-test; To evaluate whether there was a statistically significant difference in the ability to return to the original color at the SIDE point (Eab*S4) between the AG and EG groups, using the Welch's t-test. A p-value 0.05 was considered the threshold for statistical significance in this study.

## Results

The means and standard deviations of all Eab* values are presented in Table 3.

Table 3

**Figure 3:**
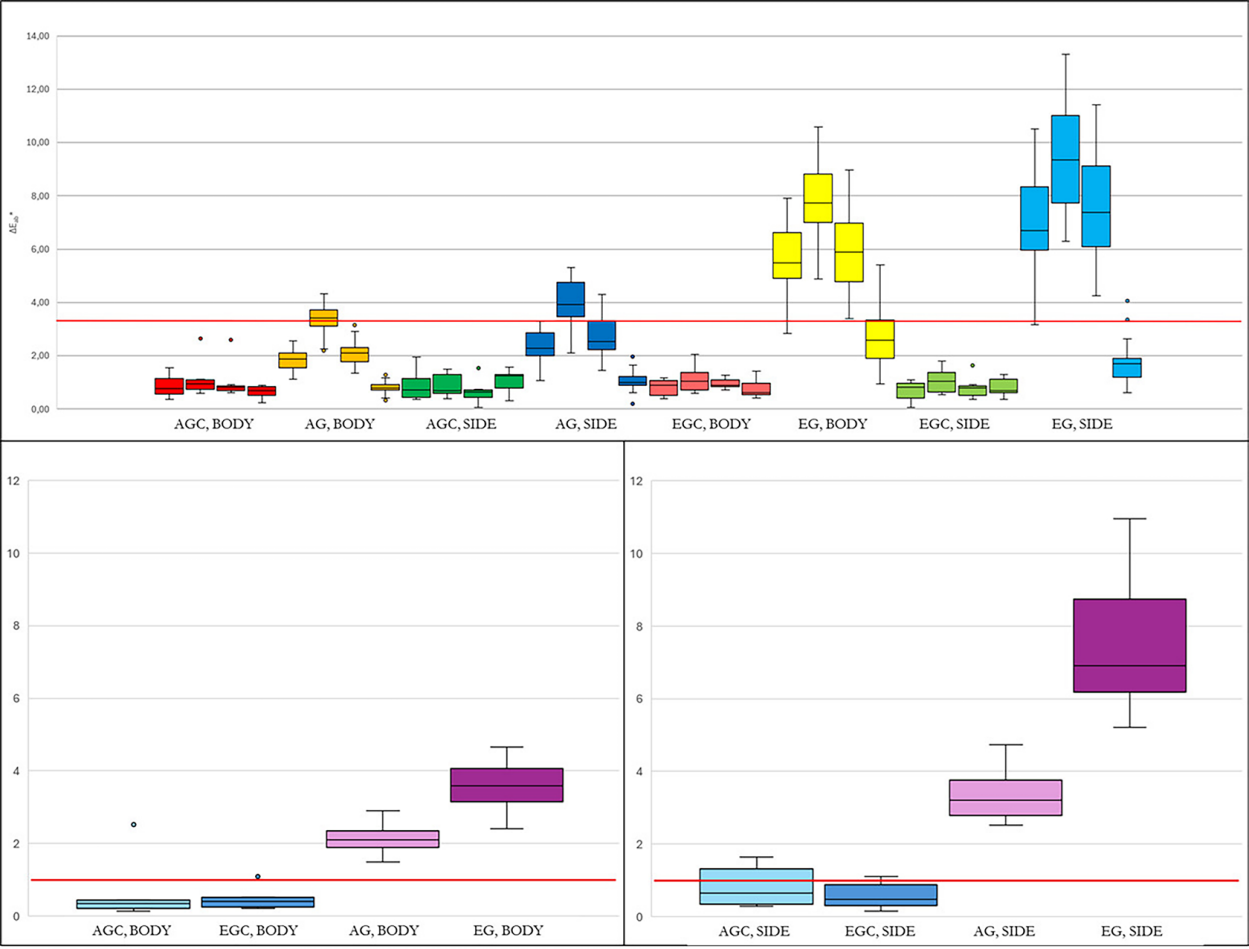
Legend: Box-and-whisker plots of all ΔEab values. In the top plot, the color of each box identifies the group and the measurement point.

From a preliminary analysis of the data, it was observed that nearly all Eab* values in the AGC and EGC groups were below 1, indicating a color variation under the threshold of visual perceptibility to the human eye (6). Consequently, the bleaching effectiveness in both control groups was also represented by a Eab*5 &lt; 1. This finding is consistent with the study's methodology, as the control specimens were immersed in physiological solution, a pigment-free medium. All Eab* values are graphically represented as box-and-whisker plots in Fig. 3.

3

These color sets are repeated four times, corresponding-from left to right-to Eab at T1, T2, T3, and T4 relative to T0; the horizontal red line marks Eab* = 3.3. The lower plots display Eab* values at the BODY point (left) and the SIDE point (right). In both lower plots, the horizontal red line indicates Eab* = 1.). Analysis of the first plot showed that the specimens in both control groups maintained a constant color throughout the entire experiment, both at the BODY and SIDE points, even after the whitening protocol. In contrast, all study groups exhibited a progressive color change at both BODY and SIDE points. An initial variation appeared at T1, followed by an increase in color difference at T2, and a subsequent reduction at T3, approaching Eab* values similar to those recorded at T1. Specimens in both AG and EG groups showed color variations above the clinical acceptability threshold (Eab* &gt; 3.3) at T2. In the AG group, Eab* values decreased below 3.3 at T3, whereas in the EG group this occurred only at T4, after the whitening protocol. After the whitening phase, none of the groups showed mean Eab*4 values exceeding 3.3: AGC, Eab*B4: 0.65 (95% CI: 0.42-0.87); EGC, Eab*B4: 0.79 (95% CI: 0.46-1.11); AG, Eab*B4: 0.82 (95% CI: 0.71-0.93); EG, Eab*B4: 2.74 (95% CI: 2.21-3.26); AGC, Eab*S4: 1.05 (95% CI: 0.64-1.45); EGC, Eab*S4: 0.83 (95% CI: 0.51-1.15); AG, Eab*S4: 1.07 (95% CI: 0.91-1.23); EG, Eab*S4: 1.77 (95% CI: 1.43-2.11). Analysis of the second and third plots revealed that the bleaching effectiveness in the study groups, at both measurement points, was greater than the Eab* threshold visible to the naked eye (Eab* &gt; 1) (6). Therefore, the 40% hydrogen peroxide bleaching treatment consistently produced a visible color improvement, albeit of modest magnitude. According to the Wilcoxon signed-rank test (Table 4), a statistically significant difference between the BODY and SIDE measurement points was found in both study groups for bleaching effectiveness (AG (n=23, W=0.0, p&lt;0.001, effect size=-1.000), EG (n=23, W=0.0, p&lt;0.001, effect size=-1.000)) and in the ability to return to the original color (AG (n=23, W=56.0, p=0.011, effect size=-0.594), EG (n=23, W=227.0, p=0.005, effect size=0.645)), whereas no significant difference was observed in the control groups.

Table 4

The Welch's t-test revealed statistically significant differences in both bleaching effectiveness (t=-11.5, df=27.1, p&lt;0.001, effect size=-3.39) and color recovery ability (t=-3.81, df=31.1, p&lt;0.001, effect size=-1.12) at the SIDE point between the AG and EG groups.

## Discussion

The present study deepens the understanding of how high-concentration hydrogen peroxide interacts with universal composite resins and how surface finishing protocols may modulate both pigment absorption and whitening diffusion. Limited recent studies suggest that both pigments and bleaching agents may be able to diffuse into the polymer resin matrix up to a depth of approximately 2.5 mm, depending on the composite formulation (11 , 19). In this experiment, bleaching was applied exclusively to surface B; therefore, hydrogen peroxide diffusion can be assumed to have occurred from surface B toward the opposite side of the rectangular specimens. Accordingly, color measurement at the SIDE point reflect the chromatic changes immediately beneath surface B, whereas measurements at the BODY point correspond to a depth of 5 mm beneath the surface B. This methodological approach allows the penetration depth of the whitening effect to be evaluated without sectioning the specimens, as the limited thickness of the samples enables the direct observation from surface A of chromatic changes beneath surface B. Based on these considerations, and the results of the present study, it could be concluded that the bleaching agent penetrates the composite structure (11). Additionally, bleaching effectiveness was higher among the EG specimens compared to AG specimens. More importantly, these findings open new perspectives on how polishing and whitening may influence the long-term esthetic management of resin-based restorations. The comparison between multi-step and single-step finishing systems revealed not only quantitative differences in color variation but, more significantly, qualitative distinctions in surface behavior. Multi-step polishing produced surfaces that were more resistant to pigment uptake and less permeable to whitening agents, whereas single-step systems showed greater susceptibility to both staining and bleaching (7 , 9 , 10). This suggests that surface topography acts as a regulator of diffusion: a rougher or more porous resin surface not only absorbs chromogens more readily but could also allow deeper penetration of oxidizing molecules. From a clinical perspective, this relationship highlights the dual role of finishing-not merely as an esthetic refinement but as a determinant of the restorative material's chemical stability over time. The demonstration that 40% hydrogen peroxide can influence color even several millimeters away from the site of application, as shown by Eab*B5 1, has notable translational implications. It indicates that the bleaching process in composite resins may not be limited to superficial layers and that, in clinical terms, whitening procedures could potentially affect the subsurface optical behavior of restorations. However, this effect may arise from both true chemical diffusion and optical blending, especially in composites such as Omnichroma that possess high color-adjustment potential (CAP) (20 , 21). This study was not designed to determine which of these two mechanisms is primarily responsible; however, distinguishing them is not merely a scientific curiosity-it is fundamental to predicting the long-term color dynamics of contemporary universal composites exposed to whitening agents. Future research employing depth-resolved spectrophotometry, micro-Raman analysis, or optical coherence tomography could clarify whether these color changes correspond to real chemical alterations or to light-transmission phenomena. From a translational standpoint, these insights call for a more individualized approach to the maintenance of composite restorations. The simulation of one year of clinical exposure within this study provides a useful temporal framework: it suggests that color alterations resulting from daily dietary habits could accumulate within a predictable timeframe and be reversed through controlled whitening and re-polishing (14 , 15). Annual maintenance, especially in patients with high intake of chromogenic beverages, could therefore prevent deep irreversible staining while maintaining material integrity. However, further studies are needed to determine the exact timeframe, as this study did not account for tooth brushing, thermal cycling, other complex oral conditions and different composite types or staining media, which may influence both pigment uptake and the surface topography of composite restorations. Integrating such preventive whitening into recall protocols could reduce the frequency of replacement procedures, lowering biological and economic costs for both patients and clinicians. The results also reinforce the relevance of surface finishing as a modifiable clinical factor. Selecting multi-step polishing systems-despite their slightly longer chair time-should be viewed as an investment in the restoration's longevity, given their ability to minimize both pigment retention and oxidative degradation (7 , 9 , 10). Conversely, single-step systems, while convenient, may require more frequent maintenance to compensate for their lower resistance to pigment diffusion. This trade-off should be considered when planning restorations in highly visible areas or in patients with strong aesthetic demands. Another important translational implication concerns the selective whitening of discolored composite restorations. Since enamel is more reactive to bleaching than resin (4), uncontrolled extension of the gel over restoration margins may accentuate color mismatch and weaken the enamel-composite interface (4 , 22). The current findings suggest that, particularly with universal composites that display strong optical blending, whitening restricted to the central area of the restoration could be able to restore esthetic harmony without exposing enamel to the bleaching agent (21). In practice, this could simplify protocols, shorten treatment times, and preserve marginal adhesion, thereby supporting the conservative philosophy of modern restorative dentistry. Beyond its clinical implications, this study highlights several research directions with direct relevance to materials science. The relationship between surface microtopography, molecular diffusion, and optical behavior remains largely unexplored. Understanding how filler morphology, resin cross-link density, and polishing methods collectively influence permeability could guide the design of next-generation composites that combine esthetic versatility with chemical resilience (17 , 19). Likewise, establishing kinetics and limits of peroxide diffusion and optical whitening could lead to optimized bleaching agents specifically formulated for restorative materials, reducing unwanted surface roughening or hardness loss (11 , 15 , 17). In summary, the present investigation demonstrates that bleaching agents can induce whitening of universal composite resins at depth and that surface finishing significantly modulates this process. More importantly, it repositions these findings within a translational context: finishing protocols should be conceived not merely as steps toward gloss, but as barriers or facilitators of molecular transport; whitening should be integrated into a preventive maintenance strategy, not used as an isolated corrective measure; and the interaction between optical and chemical phenomena should become a central focus of future dental-materials research. By reframing these results within a clinical and scientific continuum, this study contributes to a more nuanced and forward-looking understanding of how restorative materials behave under the dual challenges of pigmentation and bleaching.

## Conclusions

According to the results of the present in vitro study, it can be concluded that 40% hydrogen peroxide is able to induce whitening of the universal composite Omnichroma at depth. Moreover, the bleaching effectiveness of 40% hydrogen peroxide was greater in specimens polished with the single-step Enhance® PoGo® system compared with those polished with the multi-step Astropol® system, although the latter demonstrated superior color stability.

## Figures and Tables

**Table 1:** Technical characteristics and operating protocols of polishing systems.

Polishing system	Manufacturer	Type	Composition	Grain	Grain size	Tip shape	Speed
Astropol®	Ivoclar Vivadent AG, Schaan, Liechtenstein	2-step	Silicone rubber	Silicon carbide (Carborundum)	F (36.5 µm),P (12.8 µm)	Point	10.000 rpm
Enhance® PoGo®	Dentsply Caulk, Milford, DE, USA	1-step	Polymerized Urethane Dimethacrylate (UDMA)	Silicon dioxide, diamond	PoGo (7 µm)	Point	10.000 rpm

1

**Table 2:** Five time-points for colour measurement.

Time-point	Control groups	Study groups
T0	Before ageing in the respective solutions	
T1	After 7 days of exposure to physiological solution	After 7 days of exposure to red wine
T2	After 14 days of exposure to physiological solution	After 14 days of exposure to red wine
T3	After 24 hours of exposure to physiological solution	
T4	After whitening treatment	

2

**Table 3:** Means and standard deviations of ΔEab*B1-5 and ΔEab*S1-5 values across all groups.

ΔEab*	AGC		EGC		AG		EG	
	Mean	Std dev	Mean	Std dev	Mean	Std dev	Mean	Std dev
B1	0.87	0.47	0.81	0.32	1.82	0.37	5.56	1.21
B2	1.12	0.70	1.13	0.53	3.33	0.60	7.81	1.48
B3	1.03	0.70	0.97	0.20	2.11	0.44	5.95	1.44
B4	0.65	0.24	0.79	0.36	0.82	0.25	2.74	1.22
B5	0.62	0.84	0.46	0.30	2.10	0.35	3.63	0.61
S1	0.89	0.58	0.67	0.38	2.33	0.61	7.05	1.77
S2	0.91	0.45	1.06	0.50	4.04	0.90	9.50	1.94
S3	0.65	0.46	0.80	0.43	2.74	0.75	7.45	2.15
S4	1.05	0.44	0.83	0.34	1.07	0.37	1.77	0.80
S5	0.79	0.51	0.56	0.33	3.30	0.58	7.55	1.68

3

**Table 4:** Wilcoxon W test results for ΔEab*5 and ΔEab*4 differences between measurement points.

	Group	p-value		Group	p-value
Bleaching effectiveness	AGC	0.375	Ability to return to the original color	AGC	0.078
	AG	< 0.001		AG	0.011
	EGC	0.688		EGC	0.578
	EG	< 0.001		EG	0.005

4

## Data Availability

The datasets used and analyzed during the current study are available from the corresponding author.
